# Effect of Cold Spells and Their Different Definitions on Mortality in Shenzhen, China

**DOI:** 10.3389/fpubh.2021.817079

**Published:** 2022-01-24

**Authors:** Chengzhen Meng, Fang Ke, Yao Xiao, Suli Huang, Yanran Duan, Gang Liu, Shuyuan Yu, Yingbin Fu, Ji Peng, Jinquan Cheng, Ping Yin

**Affiliations:** ^1^Department of Epidemiology and Biostatistics, School of Public Health, Tongji Medical College, Huazhong University of Science and Technology, Wuhan, China; ^2^Children's Health Care Hospital, Wuhan, China; ^3^Shenzhen Center for Disease Control and Prevention, Shenzhen, China; ^4^Shenzhen Center for Chronic Disease Control, Shenzhen, China

**Keywords:** temperature, cold spell, characteristics, mortality, vulnerable populations

## Abstract

A high premium has been put on researching the effects of cold spells because of their adverse influence on people's daily lives and health. The study aimed to find the most appropriate definition of the cold spell in Shenzhen and quantify the impact of cold spells on mortality. Based on the daily mortality data in Shenzhen from 2013 to 2017 and the meteorological and pollutant data from the same period, we quantified the effect of cold spells using eight different definitions in the framework of a distributed lag non-linear model with a quasi-Poisson distribution. In Shenzhen, low temperatures increase the risk of death more significantly than high temperatures (using the optimal temperature as the cut-off value). Comparing the quasi-Akaike information criterion value, attribution fraction (b-AF), and attribution number (b-AN) for all causes of deaths and non-accidental deaths, the optimal definition of the cold spell was defined as the threshold was 3rd percentile of the daily average temperature and duration for 3 or more consecutive days (all causes: b-AF = 2.31% [1.01–3.50%], b-AN = 650; non-accidental: b-AF = 1.92% [0.57–3.17%], b-AN = 471). For cardiovascular deaths, the best definition was the temperature threshold as the 3rd percentile of the daily average temperature with a duration of 4 consecutive days (cardiovascular: b-AF = 1.37% [0.05–2.51%], b-AN = 142). Based on the best definition in the model, mortality risk increased in cold spells, with a statistically significant lag effect occurring as early as the 4th day and the effect of a single day lasting for 6 days. The maximum cumulative effect occurred on the 14th day (all-cause: RR = 1.54 [95% CI, 1.20–1.98]; non-accidental: RR = 1.43 [95% CI, 1.11–1.84]; cardiovascular: RR = 1.58 [95% CI, 1.00–2.48]). The elderly and females were more susceptible to cold spells. Cold spells and their definitions were associated with an increased risk of death. The findings of this research provide information for establishing an early warning system, developing preventive measures, and protecting susceptible populations.

## Introduction

Extreme atmospheric events have increased in frequency, intensity, and severity during the last several decades as a result of global climate change (1, 2). Because of the impact of severe temperature occurrences on people's everyday lives and health, a premium has been placed on describing and investigating their effects. Numerous studies have demonstrated that extra-temperature factors significantly impact mortality and morbidity (3, 4). The effect relationship is U- or inverse J-shaped, and there is a threshold effect at which the fatality risk increases at above or below the optimal temperature (5–7). However, the optimum temperature and its associated health effects vary across cities, countries, and areas (8–10). Particularly concerning is the fact that even during periods of global warming, extreme cold temperatures continue to increase, with a stronger tendency for cold spell occurrences (11, 12).

The cold spell is a distinctive type of extreme atmospheric event that manifests as anomalous low-temperatures over consecutive days (13, 14). The detrimental effects of heatwaves have been extensively researched, little knowledge exists about the impacts of cold spells on mortality in temperate regions. Exposure to a cold spell increases the risk of premature morbidity and mortality, especially for patients with cardiovascular and respiratory diseases, and for older adults (15–17).

Currently, there is no standard definition of a cold spell. In Chinese meteorology, the definition of a cold spell consists of three scenarios. After the transit of cold air in an area, (i) the minimum temperature drops by more than 8°C within 24 h; (ii) the temperature drops by more than 10°C within 48 h; (iii) the temperature drops by more than 12°C continuously within 72 h, with the minimum temperature below 4°C simultaneously (http://www.cma.gov.cn). These definitions are based on absolute temperatures and do not take regional variances into account. As a result, the majority of studies described cold spells using the percentile threshold approach, which considers two indications: the temperature threshold and the duration of the temperature below the thresholds (15, 18). This method provides an objective and representative assessment of the effect of a cold spell. Although this conforms to the generally recognized definition of a cold spell, there are minor variations in the definition of cold spells among researchers. Zhou et al. utilized the 5th percentile of daily mean temperatures as an indication of exposure with a duration of at least 5 consecutive days between December and March in China (19). In the Czech Republic, a cold spell was defined as at least 2 consecutive days with average daily temperatures below the 10th percentile of the mean annual cycle (20). This discrepancy in definitions might be due to regional variation.

The effect of the cold spell on human health has been examined extensively in developed countries. However, few studies have been performed in developing countries. Investigating the association between cold spells and human health is critical for developing countries, which lag behind developed countries in their capacity to manage severe climatic events. In addition, China is a vast territory with diverse temperatures, economic growth, and lifestyles. Using the definition in extant research does not adequately represent the effect of the cold spell in China (21–23). Hence, the definitions and impacts of cold spells varied according to the locations and demographic groups examined in the studies. It becomes essential to explore the optimal definition of cold spell, to analyze its influences, and to create suitable preventive measures in a given location.

In the present study, we aimed to determine the most appropriate definition of a cold spell in Shenzhen by evaluating eight different definitions. Then, using the most accurate definition, the effect of cold spells on mortality was investigated. Finally, a subgroup analysis was conducted to identify the vulnerable populations.

## Materials and Methods

### Study Area

Shenzhen is a developed coastal city in southeastern China, lying between 113°46′~114°37′ E and 22°27′~22°52′N, next to Hong Kong. The city has a subtropical monsoon climate with year-round southeast winds and extended periods of sunlight. Each year, the rainy season lasts from April until September. In other months, the weather is dry and warm. Compared to other central and northern China cities, Shenzhen has better air quality as China's first special economic zone. As of December 2017, the resident population was 12.53 million.

### Data Sources

Daily meteorological data for Shenzhen were acquired from the Shenzhen Meteorological Service Center from 2013 to 2017, including daily temperature (°C) and relative humidity (%). Air pollution data, including daily average concentrations of ozone (O_3_), carbon monoxide (CO), nitrogen dioxide (NO_2_), inhalable particles with aerodynamic diameter <10 mm (PM10), inhalable particles with aerodynamic diameter <2.5 mm (PM2.5), and sulfur dioxide (SO_2_), were measured by seven national monitoring stations in Shenzhen. Extreme cold, cold, mild cold, mild heat, heat, and extreme heat were classified according to specific percentile (1st, 5th, 25th, 75th, and 95th). Daily mortality data were provided by the Shenzhen Center for Disease Control and Prevention (CDC). The cause of death was coded and classified by the Classification of Diseases 10th version (ICD-10) as follows: all-causes (ICD-10: A00-U99); non-accidental (ICD-10: A00-R99); cardiovascular diseases (ICD-10: I00-I99) and respiratory disease (J00-J99); accidental (ICD-10: V01-X59). Daily non-accidental and cardiovascular mortality were stratified by sex (female and male) and age (<65 and ≥65 years).

### Definition of the Cold Spell

According to studies on the health impacts of cold spells, the temperature intensity and duration need to be considered in the definition. We estimated the short-term influence of cold spells on mortality in Shenzhen considering eight types of cold spells with distinct definitions. The optimal definitions were determined by the quasi-Akaike information criterion (QAIC) (24), attribution risk fraction, and attribution risk number (25). Using daily average temperature as an exposure metric for cold conditions, which accurately captures exposure throughout the day and night, policymakers may easily comprehend the data (26). The eight different definitions of cold spells are defined as the threshold (3rd and the 5th percentile of temperatures) for at least 2, 3, 4, and 5 consecutive days in Shenzhen.

### Statistical Analysis

#### Examining the Effect of Temperature

A cross-basis function represents the non-linear exposure-outcome relationship of the delay in the distributed lag non-linear model (DLNM) (27). Previous studies showed that death has a significant non-linear relationship with the daily mean temperature. Considering that the number of daily deaths is over-dispersion, a quasi-Poisson regression model was used. The model is described as:


(1) $$\begin{array}{l} \begin{array}{ccc} log[E(Y_{t})] & = & \alpha +\beta Temp_{t,l}+NS(RH_{t},2)+NS(Time,7) \end{array}\\ \;\begin{array}{c} +\gamma HOL+\varepsilon DOW \end{array} \end{array}$$

where *t* is the observation day; *E(Y*_*t*_*)* denotes the expected count of death on day *t*; α is the intercept; and *Temp*_*t, l*_ is the matrix obtained by applying the temperature with the number of lag days *l* and the coefficient β in DLNM. We use a 4 *df* natural cubic spline function to estimate the lag effect. According to previous studies and to fully capture the total impact of temperature exposure in this region, the lag is 14 days. *NS* in the model is the natural cubic spline function; *RH*_*t*_ represents the relative humidity on day *t* with the degrees of freedom (2 *df* ); *Time* is included to control long-term temporal trend (7 *df* per year); Day of Week (*DOW*, serial number from 1 to 7) is a multi-categorical variable that indicates day t as a day from Monday to Sunday to control for the confounding effects of the day of the week; Holiday (*HOL, HOL* = 1 when it is a public holiday) is a binary variable that denotes a legal holiday in China to control the confounding effect. ε and γ are the coefficients. The choices of the degrees of freedom were optimized with QAIC.

#### Examining the Effect of the Cold Spell

Data from November to March of each year were included from the time-series change-point analyses to prevent any potential bias due to heat effects and the lag effects of a cold spell on daily death. To investigate the impact of a cold spell on different lags and its cumulative effect on mortality, a DLNM with a quasi-Poisson distribution was constructed. The model using eight different definitions of cold spells is:


(2) $$\begin{array}{l} \begin{array}{ccc} log[E(Y_{t})] & = & \alpha +\beta_{1}CS_{t,l}+NS(Temp_{t},2)+NS(RH_{t},2) \end{array}\\ \;\begin{array}{c} +NS(Time,7)+\gamma HOL+\varepsilon DOW \end{array} \end{array}$$

where *CS*_*t*_ refers to the dummy variable that indicates the cold spell on day *t* (0: days without cold spell; 1: days with cold spell); β_1_*CS*_*t,l*_ is the cross-basis object to estimate the effect of cold spells; β_1_ is vectors of the regression coefficients of the cold spell, with a linear function of exposure-response dimension, and the lag effect of the cold spell is a natural cubic spline function of 4 *df* ; the maximum lag of the cold spell is set as 14 days.

Compared with RR, attribution fraction and number can better quantify the disease burden caused by a cold spell, which is more instructive for developing public health interventions. Under the DLNM framework, the attributable risk fraction and number are calculated from a backward perspective. The 95% empirical confidence intervals (eCI) were estimated from the related 2.5 and 97.5 percentiles of the resultant distribution through Monte Carlo simulation. Additionally, stratified analyses were also conducted by age group (<65 or ≥65 years) and sex (female and male).

We tested for statistically significant differences in effect estimates between the categories of the potential effect modifiers (e.g., between males and females) by calculating the 95% confidence intervals (95% CI):


(3) $$\begin{array}{r} \left({\hat{Q}}_{1}-{\hat{Q}}_{2}\right)\pm 1.96\sqrt{{\hat{SE}}_{1}^{2}+{\hat{SE}}_{2}^{2}} \end{array}$$

where ${\hat{Q}}_{1}$ and ${\hat{Q}}_{2}$ are the estimates for the two categories, and ${\hat{SE}}_{1}$and ${\hat{SE}}_{2}$ are their respective standard errors (28, 29).

#### Sensitivity Analysis

We evaluated the robustness of the study results by varying the *df* of time (6 and 8 per year), varying the *df* of cold spells (3 and 5), varying the *df* of average daily temperature and relative humidity (3 and 4), and adding air pollution to the model. All statistical analyses were performed in R software (version 3.5.2) using the dlnm package (30).

## Results

### Characteristic Causes of Death

Table 1 displays the number of total deaths from 2013 to 2017 in Shenzhen. The number of deaths was slightly increased year by year, of which a large proportion of deaths causes were non-accidental. The daily mortality from non-accidental and cardiovascular disease, stratified by sex and age, is summarized in Supplementary Table S1. There were 65,325 all-causes of deaths, 56,034 non-accidental deaths, and 23,030 cardiovascular deaths in the whole study. In non-accidental deaths, 61.90% were males and 54.79% were in the elderly. For cardiovascular deaths, 61.60% were males, and 64.10% were in the elderly. The average daily number of non-accidental deaths was 30.72. Among them, the number of deaths in the <65 age groups was 16.83, significantly more than that in the ≥65 age groups.

**Table 1:** Summary statistics for mortality in Shenzhen, 2013–2017.

**Causes of death**	**Period**					
	**All**	**2013**	**2014**	**2015**	**2016**	**2017**
All causes	65,325	11,936	12,847	12,998	14,476	13,068
Non-accidental	56,034	10,161	10,920	11,206	12,328	11,419
Cardiovascular	23,030	4,654	4,628	4,602	4,733	4,413
Respiratory	4,197	626	658	727	1,137	1,049
Other^a^	29,433	5,507	5,634	5,877	6,458	5,957
Accidental	5,400	1,035	1,136	991	1,301	937
Other^b^	3,891	740	791	801	847	712

a *“Other” meant other causes of death that do not include cardiovascular cause and respiratory cause in the non-accidental death category*.

b *“Other” meant other causes of death that do not include non-accidental cause and accidental cause in the all causes death category*.

### Impact of Temperature and Mortality

**Figure 1:**
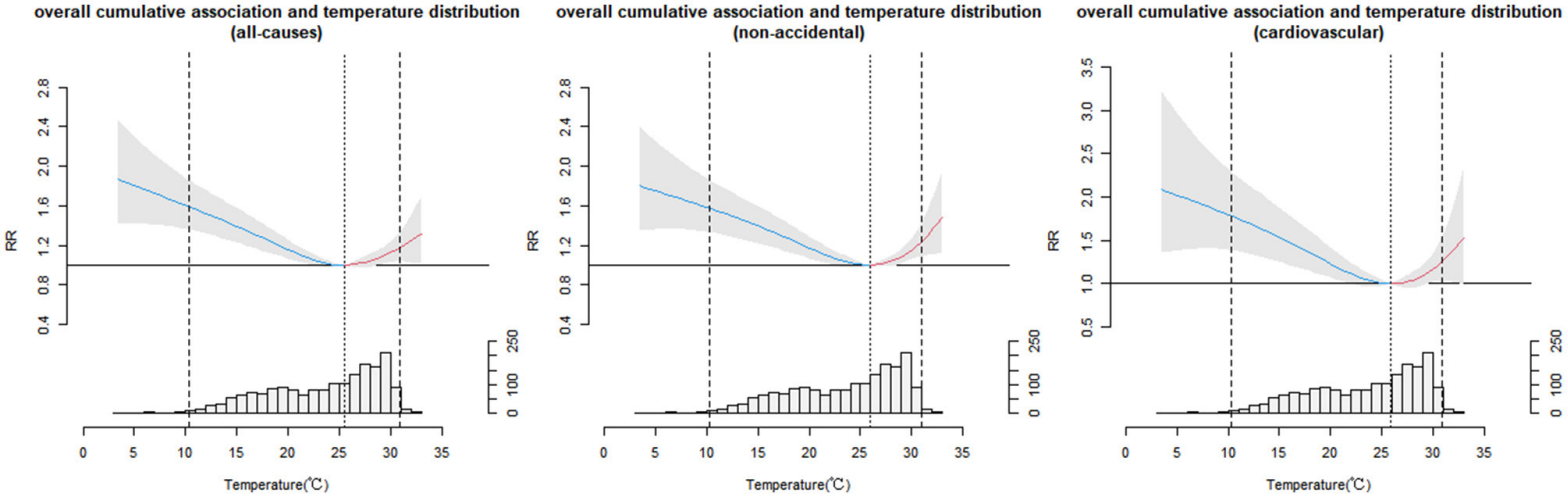
Overall cumulative exposure-response relationship between temperature distribution and mortality in Shenzhen, 2013–2017.

Figure 1 shows the cumulative exposure-response effect of temperature on total deaths, non-accidental deaths, and deaths from cardiovascular diseases. The curves for all three exposure effects were U-shaped, where the lowest adverse effect temperature was 25.5°C for the all-causes of deaths, 26°C for the non-accidental deaths, and 25.8°C for the cardiovascular deaths. Both below and above optimal temperature increase the risk of death, and the effect is statistically significant. The low-temperature effect was more significant than the high-temperature effect for all three outcomes (using the optimal temperature as the reference value). The complete results are listed in Supplementary Table S2.

### Impact of Cold Spell and Mortality

The total number of days that a cold spell event occurred during the study period varied with the definition of a cold spell (Table 2). For example, under the lenient criteria, when the threshold was below the 5th percentile of the daily average temperature with a duration of ≥2 days, the total number of days was 83 days. The total number of days only was 12 days under the stringent definition, when the threshold was set as the temperature below the 3rd percentile of the daily average temperature last for ≥5 consecutive days.

**Table 2:** The QAIC value, attributable risk fraction, and attributable risk number calculated in the model under the eight different definitions of the cold spell.

**Percentile**	**No. of consecutive days**	**Cold spell days**	**All-causes**			**Non-accidental**			**Cardiovascular**		
			**QAIC**	**b-AF (%)**	**b-AN (*n*)**	**QAIC**	**b-AF (%)**	**b-AN (*n*)**	**QAIC**	**b-AF (%)**	**b-AN (*n*)**
≤ 3rd	≥2	49	5105.86	2.09 (0.65–3.49)	591	4894.57	1.64 (0.09–3.07)	405	4215.01	1.74 (−0.63 to 3.88)	181
	≥3	41	5104.09	2.31 (1.01–3.50)	650	4892.65	1.92 (0.57–3.17)	471	4214.82	1.66 (−0.51 to 3.58)	173
	≥4	20	5113.84	1.02 (0.15–1.78)	287	4895.01	1.06 (0.19–1.83)	260	4206.87	1.37 (0.05–2.51)	142
	≥5	12	5107.16	0.92 (0.40–1.37)	258	4891.13	0.88 (0.34–1.34)	215	4211.29	0.83 (−0.04 to 1.53)	86
≤ 5th	≥2	83	5104.29	2.28 (0.13–4.26)	644	4890.76	2.12 (−0.06 to 4.13)	521	4259.48	0.87 (−1.45 to 2.95)	91
	≥3	71	5105.75	2.28 (0.44–4.04)	643	4894.66	1.94 (0.04–3.65)	476	4216.70	1.09 (−2.03 to 3.83)	114
	≥4	44	5114.95	1.36 (−0.22 to 2.73)	383	4895.99	1.44 (−0.16 to 2.86)	354	4216.25	0.66 (−2.09 to 2.91)	69
	≥5	28	5115.85	0.81 (−0.20 to 1.72)	229	4897.32	0.85 (−0.23 to 1.79)	208	4214.88	0.18 (−1.75 to 1.68)	18

*QAIC, quasi-Akaike information criterion; b-AF, attributable risk fraction; b-AN, attributable risk number*.

Because of different definitions of cold spells, even the QAIC values, attributable risk fraction, and attributable risk number calculated by the same model are not equal (Table 2). For all causes of deaths and non-accidental deaths, the optimal definition of a cold spell was the temperature threshold at the 3rd percentile of the daily average temperature with a duration of 3 consecutive days. With that definition, the QAIC value is minimum, while both b-AF and b-AN achieve their maximum values (all-cause: b-AF = 2.31% [1.01–3.50%], b-AN = 650; non-accidental: b-AF = 1.92% [0.57–3.17%], b-AN = 471). For cardiovascular deaths, the optimal definition was the temperature threshold at the 3rd percentile of the daily average temperature, with a duration of 4 consecutive days. Under the optimal definition, the QAIC values were the lowest, while the b-AF value and b-AN value were the highest, and all of them were statistically significant (cardiovascular: b-AF = 1.37% [0.05–2.51%], b-AN = 142).

Subsequent analysis was performed based on the best definition and optimal model. The cumulative lag effect of cold spells with the optimal definition is displayed in Table 3. Mortality risk increased with the cold spell in all three outcomes, with a statistically significant lag effect occurring as early as the 4th day and the effect of a single day lasting up to a total of 6 days. Moreover, the maximum cumulative effect occurred on the 14th day (all-cause: RR = 1.54 [95% CI, 1.20–1.98]; non-accidental: RR = 1.43 [95% CI, 1.11–1.84]; cardiovascular: RR = 1.58 [95% CI, 1.00–2.48]).

**Table 3:** Summary of cumulative relative risk of the cold spell on mortality at lag 0–14 days under the best definition.

**Group**	**Cumulative relative risk (RR, 95% CI)^a^**				
	**Lag 0**	**Lag 0–3**	**Lag 0–7**	**Lag 0–10**	**Lag 0–14**
All-causes	1.00 (0.96–1.05)	1.08 (0.96–1.21)	1.25 (1.07–1.46)*	1.36 (1.11–1.65)*	1.54 (1.20–1.98)*
Non-accidental	1.00 (0.95–1.05)	1.06 (0.94–1.19)	1.20 (1.02–1.41)*	1.29 (1.05–1.57)*	1.43 (1.11–1.84)*
Females	1.02 (0.95–1.10)	1.10 (0.92–1.32)	1.24 (0.97–1.58)	1.39 (1.02–1.88)*	1.55 (1.05–2.27)*
Males	0.99 (0.93–1.05)	1.03 (0.90–1.20)	1.18 (0.97–1.44)	1.23 (0.96–1.57)*	1.36 (1.00–1.86)*
<65 years	1.01 (0.95–1.32)	1.09 (0.92–1.29)	1.23 (0.98–1.54)	1.32 (1.00–1.76)*	1.46 (1.02–2.10)*
≥65 years	0.99 (0.93–1.06)	1.04 (0.89–1.21)	1.18 (0.96–1.46)*	1.26 (0.97–1.64)*	1.40 (1.01–1.96)*
Cardiovascular	1.03 (0.94–1.14)	1.22 (0.98–1.51)	1.48 (1.13–1.95)*	1.52 (1.07–2.16)*	1.58 (1.00–2.48)*
Females	1.08 (0.93–1.25)	1.36 (0.97–1.90)	1.81 (1.18–2.77)*	2.11 (1.22–3.66)*	2.35 (1.16–4.76)*
Males	1.00 (0.89–1.14)	1.13 (0.86–1.48)	1.30 (0.92–1.83)	1.21 (0.77–1.89)^†^	1.19 (0.67–2.12)^†^
<65 years	1.16 (0.99–1.36)	1.48 (1.04–2.11)*	1.49 (0.96–2.33)	1.41 (0.78–2.53)	1.27 (0.59–2.73)
≥65 years	0.97 (0.87–1.09)	1.09 (0.84–1.42)	1.46 (1.05–2.03)*	1.55 (1.02–2.37)*	1.72 (1.00–2.95)*

a *The results of cumulative relative risk at lag 0–14 days were performed under the best definition in model*.

* *Statistically significant results at the 5% level (P < 0.05)*.

† *Z-test for the difference between the two relative risks of subgroup analysis results at the 5% level (P < 0.05)*.

### Impact of Cold Spells Stratified by Sex and Age

**Figure 2:**
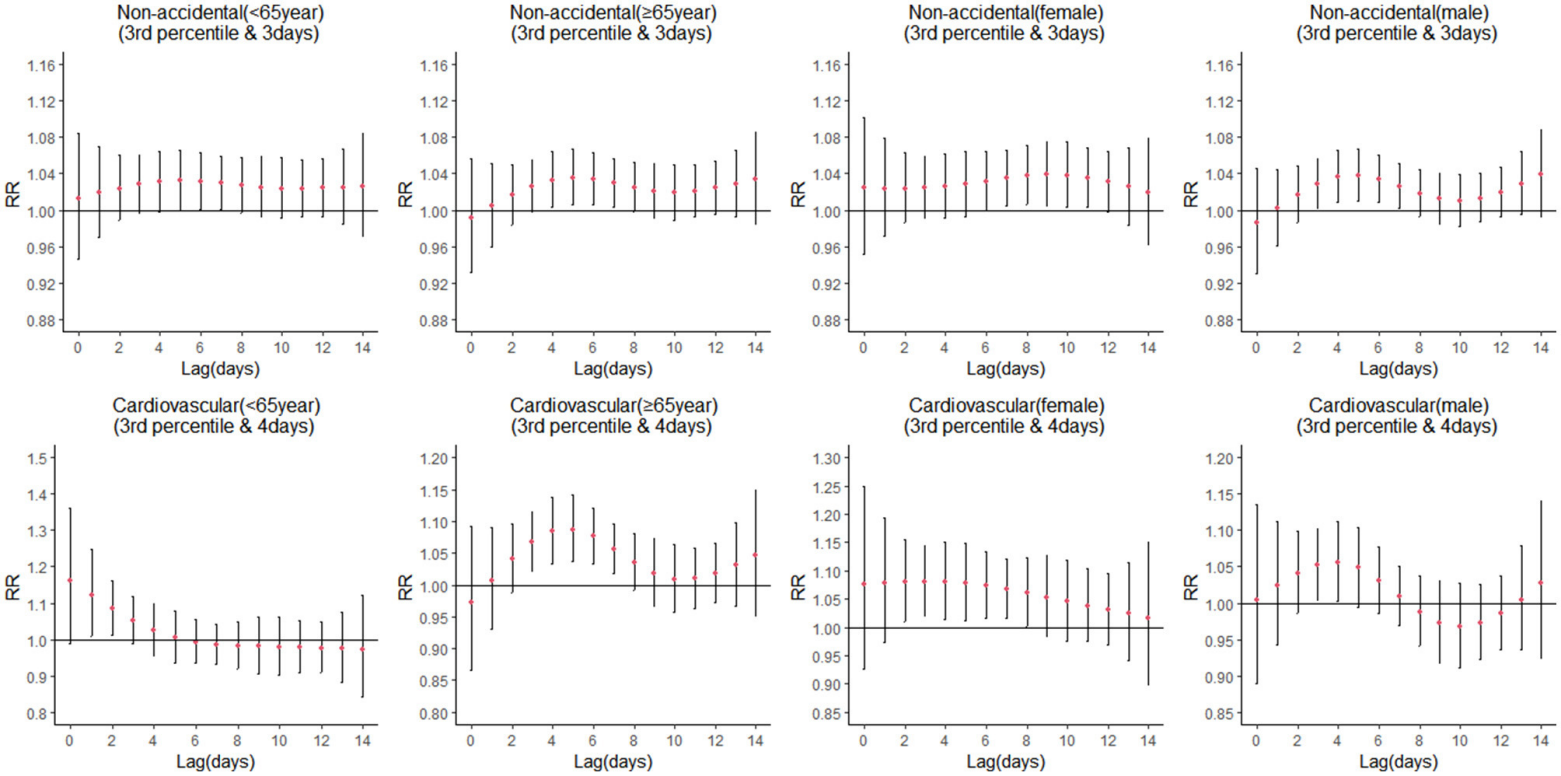
Lag-effects of the cold spell on mortality (non-accidental and cardiovascular) stratified by sex and age.

Stratification analyses were conducted according to sex and age in non-accidental and cardiovascular deaths. The cumulative lag effect and single-day effect of the cold spell on non-accidental death and cardiovascular death for each subgroup are presented in Table 3 and Figure 2, respectively. The single-day effect of the cold spell on non-accidental deaths in older people occurred on lag 5 and lasted for 3 days, with a significant cumulative effect occurring on day 14 (non-accidental: elderly: RR = 1.40 [95% CI, 1.01–1.96]). For cardiovascular deaths, the cold spell showed a more significant risk influence in females than males. The lag effect starting from lag 3 lasted for about 6 days, with the maximum cumulative RR value on the 14th day (cardiovascular: female: RR = 2.35 [95% CI, 1.16–4.76]). The effect of the cold spell on cardiovascular deaths in the elderly appeared at lag 4 and lasted for about 5 days, with the maximum cumulative RR value on the 14th day (cardiovascular: elderly: RR = 1.72 [95% CI, 1.00–2.95]).

The non-accidental deaths subgroups did not differ significantly on attribution risk fraction and attribution risk number, and the male group value was not statistically significant (Table 4). The attributable risk fraction of cardiovascular deaths was highest for women and older people (cardiovascular: female: b-AF = 2.56% [95% eCI, 0.50–4.18%], b-AN = 104; elderly: b-AF = 1.70% [95% eCI, 0.05–3.06%], b-AN = 115).

**Table 4:** The attributable fraction and number of non-accidental and cardiovascular mortality stratified by sex and age.

**Group**	**b-AF (%)**	**b-AN (***n***)**
Non-accidental		
Females	2.39 (0.29–4.23)	225
Males	1.65 (−0.05–3.15)	249
<65 years	1.95 (0.03–3.61)	211
≥65 years	1.91 (0.05–3.55)	262
Cardiovascular		
Females	2.56 (0.50–4.18)	104
Males	0.57 (−1.26–2.06)	36
<65 years	0.88 (−1.46–2.62)	32
≥65 years	1.70 (0.05–3.06)	115

*b-AF, attributable risk fraction; b-AN, attributable risk number*.

### Sensitivity Analysis

The result showed that the model was robust, and there was no change in the conclusions by changing the temperature *df* (3–4), relative humidity *df* (3–4), cold spell *df* (3–5), time *df* (6–8), and pollutant variables (SO_2_, NO_2_, CO, O_3_, and PM2.5) (Supplementary Figures S2–S6).

## Discussion

The eight definitions of a cold spell proposed in our study encompass all combinations of two temperature thresholds (3rd and 5th percentiles) and four types of duration (2, 3, 4, and 5 days). Compared to QAIC criterion and the attribution risk fraction under eight definitions, in Shenzhen, the optimal definitions for all-cause deaths and non-accidental deaths were determined to be least 3 or more consecutive days in which the daily average temperature less than the threshold was at the 3rd percentile of daily mean temperatures. For cardiovascular deaths, the definition was at least 3 or more consecutive days in which the daily average temperature less than the threshold was at the 4th percentile of daily mean temperatures. Using the best definition, we have estimated the effect of local cold spells on all causes of death, non-accidental deaths, and cardiovascular deaths in Shenzhen and identified susceptible persons. This is the first study in the Shenzhen region to examine the most appropriate definition of a cold spell and the relationship between cold spells and mortality for different outcomes.

We have observed that temperature had an influence on mortality (Figure 1). Extreme cold, cold, mild cold, heat, and extreme heat had significant risk effects on the three outcomes, and the impacts of cold temperature were more significant than those of the heat (Supplementary Table S2). This relationship was agreed with previous results that indicated that the temperature decreases as the mortality increases (9, 31). The results confirm that cold spells increase the risk of all-cause, non-accident, and cardiovascular mortality, irrespective of which of the eight definitions are used. However, the optimal definition of a cold spell was different for the three outcomes (Supplementary Figure S1). This could also be an explanation for the different sensitivities of different death causes to low temperature (32). Among the selected optimal definitions, the lag effect of cold waves on all causes of death, non-accidental death, and cardiovascular death manifested first and lasted the longest, which has important implications for constructing an optimal early warning system. Meanwhile, there is a significant public health benefit in determining the optimal definition for an early warning system to prevent even more fatalities. According to the optimal definition, the cold spell increased the risk of all-cause and non-accidental death by 54 and 43%, respectively, and increased the risk of cardiovascular disease mortality by 58% in the cumulative lag of 14 days. Exposure to low temperatures may increase the risk of acute death events in residents with cardiovascular disease by increasing blood pressure, platelet counts, blood cholesterol, and fibrinogen levels, as well as promoting inflammatory responses, exacerbating the symptoms of cardiovascular diseases (33, 34). In winter, low temperature is an established risk factor for mortality of cardiovascular disease, as numerous previous studies had found (32, 35–38).

Sex and age were modifiers of the relationship between cold spells and mortality. Our present research discovered that during cold spells, older individuals had a greater risk of non-accidental and cardiovascular death than younger adults (Table 3). This is consistent with the findings of several studies that the risk of death in low-temperature situations is significantly greater in the elderly than in younger adults (12, 36). Because organs gradually decline with age and thermal regulation systems weaken with increasing age, and the higher incidence of chronic diseases such as respiratory, congestive heart failure and cardiovascular diseases in the elderly, along with their activity limitations, they are more susceptible to cold spell events (39). At present, China is facing severe challenges related to the aging of its population. These findings have important public health and policy implications in the current context of climate change and cold extremes. Compared with the non-cold spell period, Females had a substantial 135% increased risk of death for non-accidental causes and a significant 72% increased risk of death for cardiovascular causes on lag 14 days during the cold spell period. Furthermore, females had significantly higher cumulative lag effects of cold spells than males in non-accidental and cardiovascular deaths and had more prolonged lag effects, in line with some previous studies (40–42). This difference may be attributed to different physiology in males and females, which varies considerably (43). Men, on average, have a higher tolerance for extreme temperatures and a greater capacity to control their body temperature than women do (44). Meanwhile, females in China have a longer life expectancy than men, resulting in a larger proportion of females than males among the elderly (41). Hence, the elderly and the females need to pay special attention during cold spells.

Our study has critical public health and policy implications for Shenzhen. First, despite its low latitude, Shenzhen is more sensitive to the effects of cold spells. Previous studies have confirmed this finding, with southern regions being more susceptible to cold wave impacts than other locations (45–47). This may be because populations in warmer regions lack physiological acclimation and behavioral adaptation, making them more susceptible to cold impacts. Simultaneously, many locations lack appropriate heating systems and medical services to deal with cold periods. Second, early warning systems for cold spells play a critical role in mitigating cold-weather health hazards. A necessary precondition for designing an early warning system is a clear definition of a cold spell. Through comparing eight different definitions, we were able to determine which definition was most beneficial to prevent which cause of death. The results of our study highlight the real impact of cold spells, and the government should strengthen the development of relevant specific policies and formulate intervention programs in Shenzhen. For instance, central home heating should be provided to the cities in the south of China in winter. Prior to the onset of the cold period, the government and local communities should strengthen cold spell-related health education and awareness. Hence, maintaining an accurate and timely warning system and monitoring atmospheric parameters are essential for early warning and protecting susceptible populations. Finally, we identified populations sensitive to cold spells, emphasizing the critical nature of risk awareness and personal preventive actions. It is recommended that older adults in the southern region, especially females, avoid outside activity during cold spells. In addition, during the cold spells, we should offer more professional medical services and health education to older adults.

Nevertheless, this study has a number of limitations. First, data collection was only over a short period, 5 years. Second, the distributed lag non-linear model has been widely studied and successfully employed for investigating the effects of temperature on mortality. However, the model is more sensitive to the choice of the lag day, exhibiting a strong dependence (48). There have been studies showing that air pollutant concentrations can influence the effect of temperature on daily death rates (49–51). However, our observations did not find any significant results. Our findings do not establish a causative link between cold spells and mortality but only report associations (52). Last, the meteorological data were obtained from fixed monitoring stations, which are not a good representation of individual actual exposure levels, leading to the individual estimation errors and affecting the cold spell death effects.

## Conclusions

This study aimed to investigate the impact of cold spells, which were found to be a significant risk factor for mortality in Shenzhen. The definition of a cold spell as the threshold at the 3rd percentile of mean temperature, and duration at least 3 consecutive days, was the most appropriate for all-cause deaths and non-accidental death. For cardiovascular deaths, the optimal definition was the threshold at the 3rd percentile of mean temperature, and duration was at least 4 consecutive days. Under this definition, both older adults and women were susceptible. These findings provide a clear basis and scientific information for developing an early warning system and establishing preventive measures to reduce the health risks of the cold spell.

## Data Availability Statement

The original contributions presented in the study are included in the article/Supplementary Materials, further inquiries can be directed to the corresponding author/s.

## Author Contributions

CM and FK contributed to conception and design of the study. CM and YX analyzed the data. CM wrote the manuscript. PY and JC supervised and guided the writing of the manuscript. PY, JP, SH, YD, GL, SY, and YF conceptualized and designed the study. All authors reviewed and approved the final manuscript and contributed to the study.

## Funding

This study was supported in part by grants from the National Natural Science Foundation of China (No. 81973004) and the Fundamental Research Funds for the Central Universities (2020kfyXGYJ01).

## Conflict of Interest

The authors declare that the research was conducted in the absence of any commercial or financial relationships that could be construed as a potential conflict of interest.

## Publisher's Note

All claims expressed in this article are solely those of the authors and do not necessarily represent those of their affiliated organizations, or those of the publisher, the editors and the reviewers. Any product that may be evaluated in this article, or claim that may be made by its manufacturer, is not guaranteed or endorsed by the publisher.
