# Supplementary material for: Effect of Cold Spells and Their Different Definitions on Mortality in Shenzhen, China
Source: Front Public Health. 2022 Jan 24;9:817079. doi: 10.3389/fpubh.2021.817079 (PMC8818748; doi:10.3389/fpubh.2021.817079)
Supplement: Supplementary file 1 [file Data_Sheet_1.docx]

**Supplementary Material**

**Effect of cold spells and** **their different definitions on mortality in Shenzhen, China**

**List of Tables and Figures**

**Table S1.** Statistical characteristics for mortality stratified by sex and age from 2013 to 2017.

**Table S2.** The overall cumulative exposure-response associations between temperature and the death cause were reported as specific temperature percentiles and optimum temperature as a reference.

**Table S3.** Spearman correlation coefficients between weather parameters and air pollutant in Shenzhen, 2013-2017.

**Fig S1.** The Single day lag-effect of cold spell using the eight definitions in model.

**Fig S2.** The effects of cold spell when changing the df (3, 5) for cold spells.

**Fig S3.** The effects of cold spell when changing the df (4, 6) for time.

**Fig S4.** The effects of cold spell when changing the df (3, 4) for daily mean temperature.

**Fig S5.** The effects of cold spell when changing the df (3, 4) for relative humidity.

**Fig S6.** The effects of cold spell on death causes with controlling air pollutants.

**Table S1.** Statistical characteristics for mortality stratified by sex and age from 2013 to 2017.

| Group | Total | Mean ± SD | Min | P25 | Median | P75 | Max |
| --- | --- | --- | --- | --- | --- | --- | --- |
| All causes | 65325(100.00) | 35.71±7.71 | 3 | 31 | 35 | 40 | 76 |
| Non-accidental | 56034(100.00) | 30.72±6.85 | 2 | 26 | 30 | 35 | 70 |
| Females | 21349(38.10) | 11.70±3.79 | 1 | 9 | 11 | 14 | 28 |
| Males | 34678(61.90) | 19.01±5.03 | 1 | 16 | 19 | 22 | 45 |
| < 65 years | 25334(45.21) | 16.83±4.83 | 1 | 14 | 16 | 20 | 40 |
| ≥ 65 years | 30700(54.79) | 13.89±4.07 | 1 | 11 | 14 | 17 | 30 |
| Cardiovascular | 23030(100.00) | 12.63±4.07 | 0 | 10 | 12 | 15 | 30 |
| Females | 8843(38.40) | 4.85±2.38 | 0 | 3 | 5 | 6 | 15 |
| Males | 14187(61.60) | 7.78±3.05 | 0 | 6 | 8 | 10 | 24 |
| < 65 years | 8267(35.90) | 4.53±2.23 | 0 | 3 | 4 | 6 | 15 |
| ≥ 65 years | 14763(64.10) | 8.09±3.20 | 0 | 6 | 8 | 10 | 20 |

**Table S2.** The overall cumulative exposure-response associations between temperature and the death cause were reported as specific temperature percentiles and optimum temperature as a reference.

| Group | All causes | Non-accidental | Cardiovascular |
| --- | --- | --- | --- |
| Optimum temperature | 25.5℃ | 26℃ | 25.8℃ |
| Overall relative risk | RR (95% CI) |  |  |
| Extreme cold (1st) | 1.59(1.36-1.87) ^*^ | 1.57(1.33-1.86) ^*^ | 1.78(1.38-2.29) ^*^ |
| Cold (5th) | 1.45(1.27-1.65) ^*^ | 1.45(1.26-1.67) ^*^ | 1.61(1.30-1.99) ^*^ |
| Mild cold (25th) | 1.20(1.09-1.31) ^*^ | 1.21(1.09-1.34) ^*^ | 1.28(1.10-1.50) ^*^ |
| Mild heat (75th) | 1.05(0.98-1.12) | 1.04(0.99-1.10) | 1.04(0.95-1.14) |
| Heat (95th) | 1.13(1.03-1.24) ^*^ | 1.16(1.06-1.27) ^*^ | 1.17(1.01-1.36) ^*^ |
| Extreme heat(99th) | 1.17(1.03-1.33) ^*^ | 1.23(1.08-1.41) ^*^ | 1.26(1.02-1.54) ^*^ |

^*^Statistically significant results at the 5% level (P <0.05).

**Table S3.** Spearman correlation coefficients between weather parameters and air pollutant in Shenzhen, 2013-2017.

| Variables | Mean temperature | Relative humidity | SO2 | NO2 | CO | O3 | PM10 | PM2.5 |
| --- | --- | --- | --- | --- | --- | --- | --- | --- |
| Mean temperature | 1.000 |  |  |  |  |  |  |  |
| Relative humidity | 0.195* | 1.000 |  |  |  |  |  |  |
| SO2 | -0.089* | -0.503* | 1.000 |  |  |  |  |  |
| NO2 | -0.357* | -0.104* | 0.558* | 1.000 |  |  |  |  |
| CO | -0.413* | -0.202* | 0.298* | 0.552* | 1.000 |  |  |  |
| O3 | -0.038 | -0.316* | 0.062* | -0.210* | -0.168* | 1.000 |  |  |
| PM10 | -0.422* | -0.573* | 0.628* | 0.595* | 0.511* | 0.326* | 1.000 |  |
| PM2.5 | -0.514* | -0.502* | 0.573* | 0.590* | 0.543* | 0.326* | 0.961* | 1.000 |

Abbreviations: SO_2_, sulfur dioxide; NO_2_, nitrogen dioxide; CO, carbon monoxide; O_3_, ozone; PM_10_, particulate matter less than 10μm in aerodynamic diameter; PM_2.5_, particulate matter less than 2.5μm in aerodynamic diameter.

^*^ Statistically significant results at the 10% level (P < 0.10).


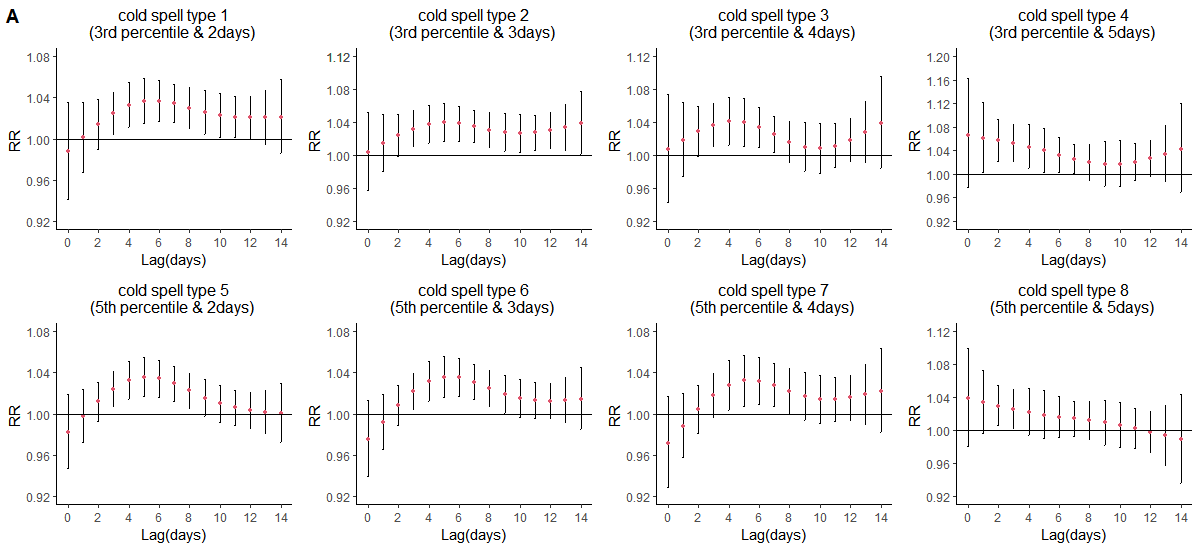


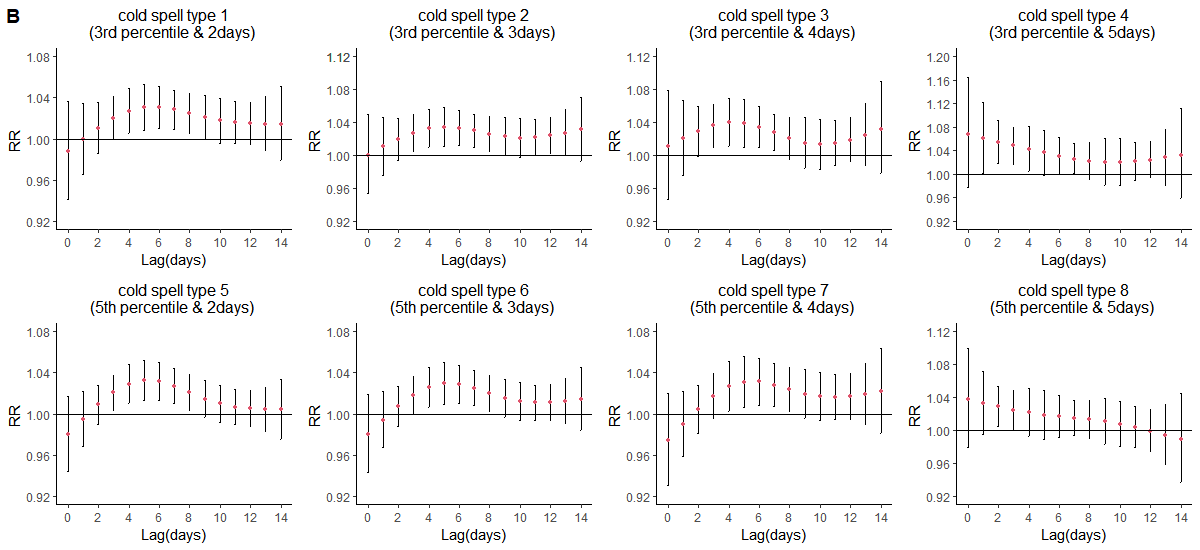


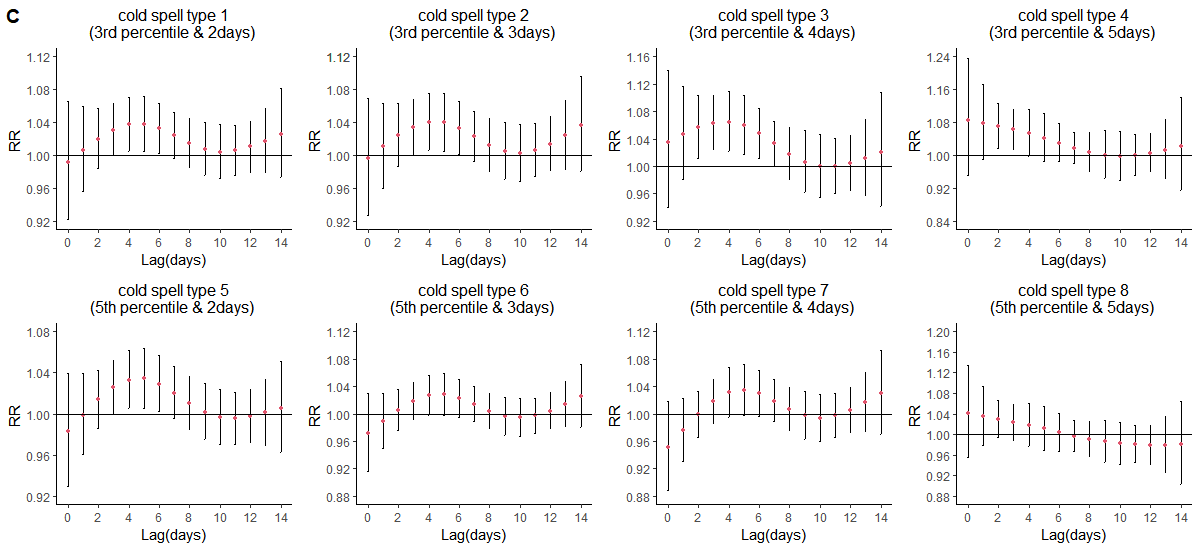


**Fig. S1.** The Single day lag-effect of cold spell using the eight definitions in model.

A: all-cause; B: non-accidental; C: cardiovascular.

**Sensitivity analysis (using the optimal definition of cold spell in model)**


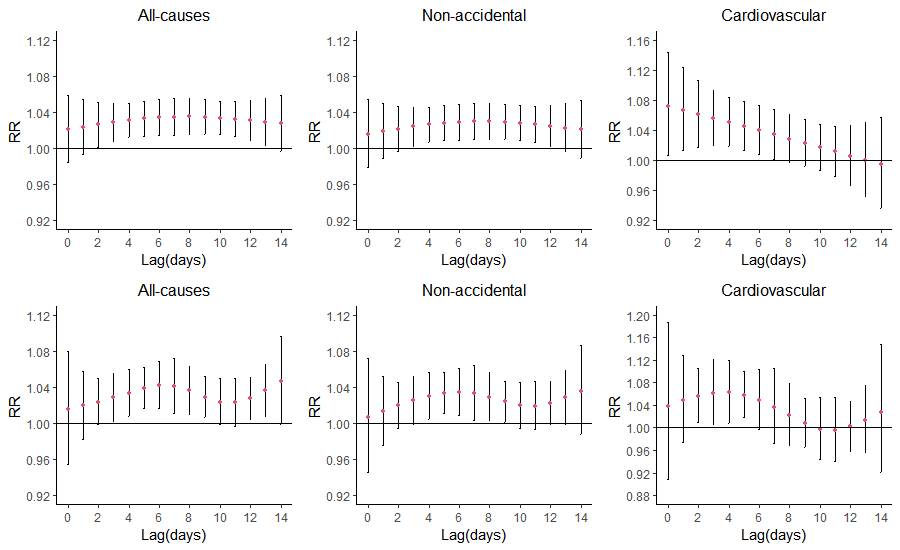


**Fig. S2.** The effects of cold spell when changing the df (3, 5) for cold spells.

Death causes: all-causes, non-accidental, cardiovascular.


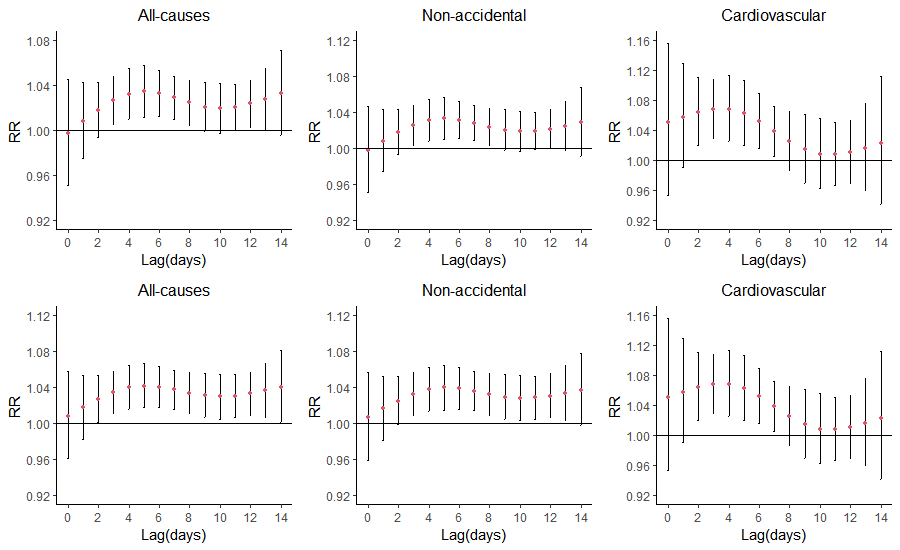


**Fig. S3.** The effects of cold spell when changing the df (6, 8) for time.

Death causes: all-causes, non-accidental, cardiovascular.


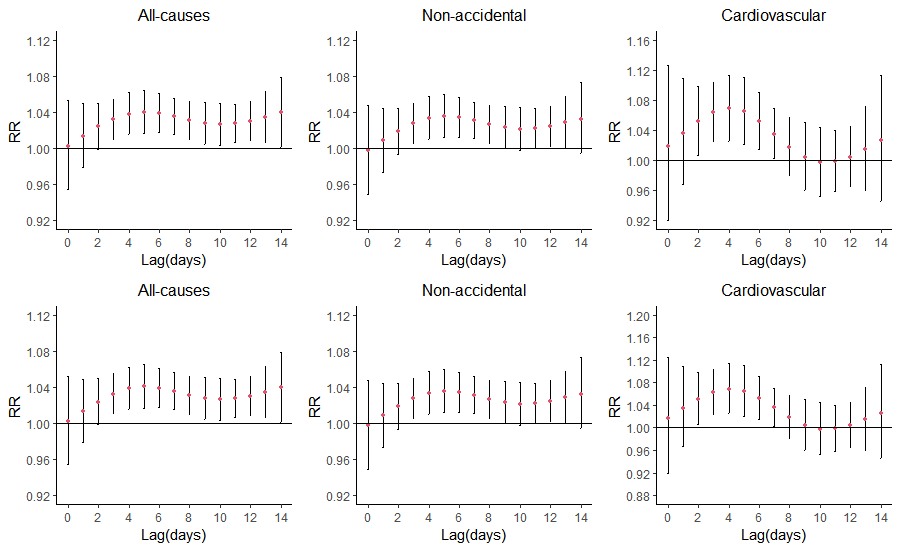


**Fig. S4.** The effects of cold spell when changing the df (3, 4) for daily mean temperature.

Death causes: all-causes, non-accidental, cardiovascular.


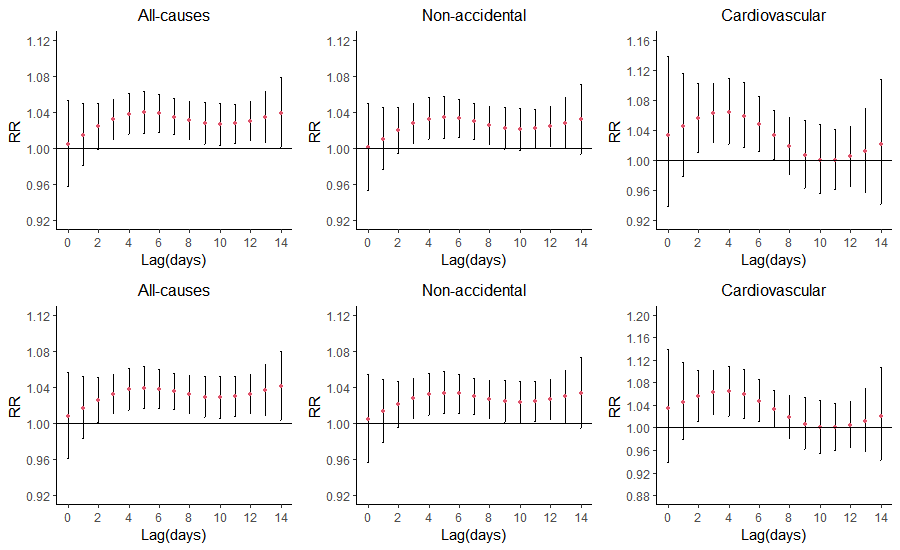


**Fig. S5.** The effects of cold spell when changing the df (3, 4) for relative humidity.

Death causes: all-causes, non-accidental, cardiovascular.


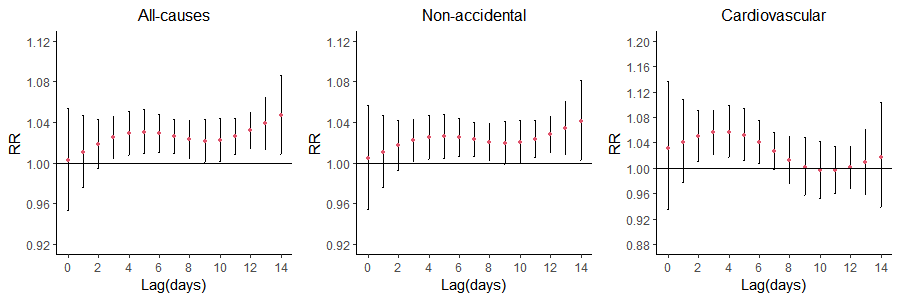


**Fig. S6.** The effects of cold spell on death causes with controlling air pollutants.

Death causes: all-causes, non-accidental, cardiovascular; controlling air pollutants: PM2.5, SO2, NO2, CO, O3.
